# Electronic Health Lifestyle Coaching Among Diabetes Patients in a Real-Life Municipality Setting: Observational Study

**DOI:** 10.2196/12140

**Published:** 2019-03-12

**Authors:** Anastasija Komkova, Carl Joakim Brandt, Daniel Hansen Pedersen, Martha Emneus, Camilla Sortsø

**Affiliations:** 1 Institute of Applied Economics and Health Research Aps Copenhagen Denmark; 2 Research Unit of General Practice Department of Public Health University of Southern Denmark Odense Denmark; 3 The Maersk Mc-Kinney Moller Institute University of Southern Denmark Odense Denmark

**Keywords:** eHealth, diabetes mellitus, healthy lifestyles, weight reduction, obesity

## Abstract

**Background:**

Internet and mobile interventions aiming to promote healthy lifestyle have attracted much attention because of their scalability and accessibility, low costs, privacy and user control, potential for use in real-life settings, as well as opportunities for real-time modifications and interactive advices. A real-life electronic health (eHealth) lifestyle coaching intervention was implemented in 8 Danish municipalities between summer 2016 and summer 2018.

**Objective:**

The aim of this study was to assess the effects associated with the eHealth intervention among diabetes patients in a real-life municipal setting. The eHealth intervention is based on an initial meeting, establishing a strong empathic relationship, followed by digital lifestyle coaching and collaboration supported by a Web-based community among patients.

**Methods:**

We conducted an observational study examining the effect of an eHealth intervention on self-reported weight change among 103 obese diabetes patients in a real-life municipal setting. The patients in the study participated in the eHealth intervention between 3 and 12 months. A weight change was observed at 6, 9, and 12 months. We used regression methods to estimate the impacts of the intervention on weight change.

**Results:**

We found that the eHealth intervention significantly reduced weight among diabetes patients, on average 4.3% of the initial body mass, which corresponds to 4.8 kg over a mean period of 7.3 months. Patients who were in intervention for more than 9 months achieved a weight reduction of 6.3% or 6.8 kg.

**Conclusions:**

This study brings forward evidence of a positive effect of a real-life eHealth lifestyle intervention on diabetes patients’ lifestyle in a municipal setting. Future research is needed to show if the effect is sustainable from a long-term perspective.

## Introduction

### Background

A majority of premature deaths from noncommunicable diseases are preventable by facilitating healthier lifestyles [1,2]. Recent systematic reviews conclude that Web-based and mobile digital electronic health (eHealth) solutions can improve lifestyle behaviors [3-8]; however, they also stress that there is a lack of “...available weight loss interventions suitable to the real-world PC setting, with most research and guideline formulation conducted inside academic silos...” [5] as well as there is a “...need for long-term interventions to evaluate sustainability” [4]. The authors have previously found that eHealth lifestyle coaching providing various behavior change techniques (BCTs) such as tailored information, self-monitoring, lifestyle coaching, in-person feedback, reminders, and person-to-person support based on a strong personal relationship led to a significant weight loss of 7.0 kg during a 20-month period [9]. A refinement of this eHealth intervention (LIVA) [10] was implemented in 8 Danish municipalities between summer 2016 and summer 2018 on the basis of a number of qualitative studies [11,12].

Municipalities invest in preventive programs with the ambition of reducing health care professional (HCP) input and time per patient while still enabling tailored and effective care [2,4,13-15]. eHealth lifestyle coaching is markedly more efficient than traditional in-person meetings [16].

In this study, we applied an observational design to investigate the first outcome data on self-reported weight change among diabetes patients participating in an eHealth intervention with municipality HCPs for at least 3 months. We combine these results with literature findings regarding the impact of weight change on diabetes patients’ costs in a municipality perspective to estimate the potential savings related to societal costs of diabetes.

The aim of this study was to evaluate data regarding self-reported weight change of eHealth lifestyle coaching among diabetes patients and assess impacts associated with offering the program as tertiary prevention among diabetes patients in Danish municipalities.

### Research Design

This is an observational study examining the effect of an eHealth lifestyle intervention on self-reported weight change among diabetes patients in a real-life municipality setting.

### Setting and Study Population

The eHealth intervention was implemented in an ongoing process in 8 Danish municipalities between summer 2016 and summer 2018. Each municipality offered the eHealth intervention within their own organizational framework with local HCPs such as dieticians, nurses, physiotherapists, and occupational therapists, with wide decision discretion resulting in a heterogeneous program setting.

The study population consisted of 103 diabetes patients obese at baseline, with body mass index (BMI) ≥30, who (1) had used the eHealth platform at any time point between June 7, 2016, and May 2, 2018, (2) had registered to use the platform because of their diabetes, (3) had at least 90 days and maximum 365 days between their first and the last weight measurement registration, and (4) had no registrations of unrealistic rapid weight change (>0.5 kg/day). All data on patients were collected from the intervention database based on patients’ own and their HCPs’ registrations.

## Methods

### Intervention

An eHealth lifestyle coaching intervention has been developed by applying the experiences from previously developed Web-based eHealth solutions used by approximately 140,000 individuals for more than 15 years on which extensive research has been conducted [6,11,16,17].

**Figure 1:**
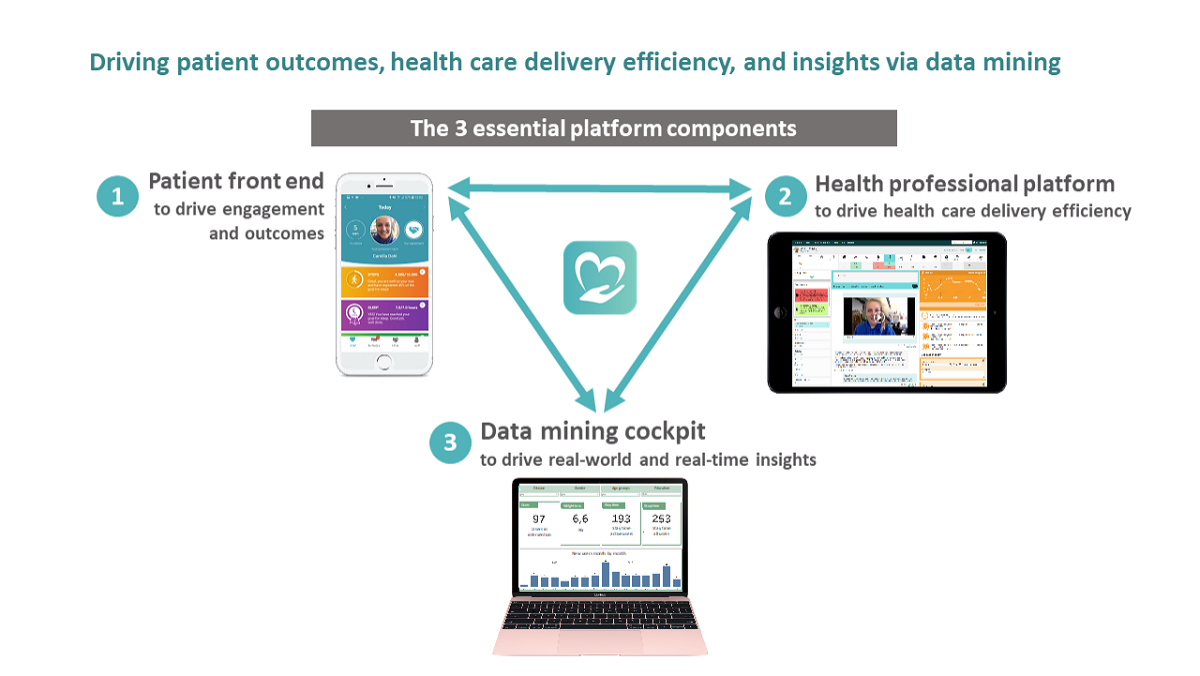
Overview of the eHealth intervention.

The key concepts in the eHealth intervention are listed in Textbox 1, and an overview is given in Figure 1.

The intervention provides various BCT’s evidenced to be effective for changing lifestyle such as tailored information, self-monitoring, lifestyle coaching, in-person feedback, reminders, and peer-to-peer support [18]. By establishing a personal relationship initially in a face-to-face meeting, which is then continued digitally through the eHealth intervention, the intervention enables tailored care and sustained patient engagement over time with a minimal of HCP input in the process of successfully changing lifestyle and sustaining this change [6,9,16].

Participants in the intervention were introduced to the eHealth intervention by a municipal HCP during a first face-to-face meeting of approximately 45 min to 60 min. Together, the participant and the municipal HCP established a relationship and agreed on goals for diet, physical exercise, sleep, and other life areas if relevant. Goal setting is based on the specific, measurable, attainable, relevant, and timely (SMART) model according to a predefined guideline structure, described in Table 1. All advices are based on recommendations from the Danish National Board of Health.

**Key concepts of the electronic health intervention.**

1. Establishment of an empathic relationship with a health care professional (HCP) in an initial face-to-face meeting.
2. Integration of main stakeholders (HCPs in the municipalities and the patients’ personal profile through smartphone or Web-based), allowing for HCPs to look over the shoulder of the patient.
3. Intuitive design enables ease of use for both the user (<1 min for registration a day) and HCPs (5-10 min per consultation) developed through ongoing extensive and systematic user involvement.
4. Different modes of communication channels allow for active communication at all levels of prerequisites among users, creating peer-to-peer support.
5. The backend control panel, including a content library and communication templates, enables optimizing of tailored quality advices asynchronously and via short message service text messaging and video.

**Figure 2:**
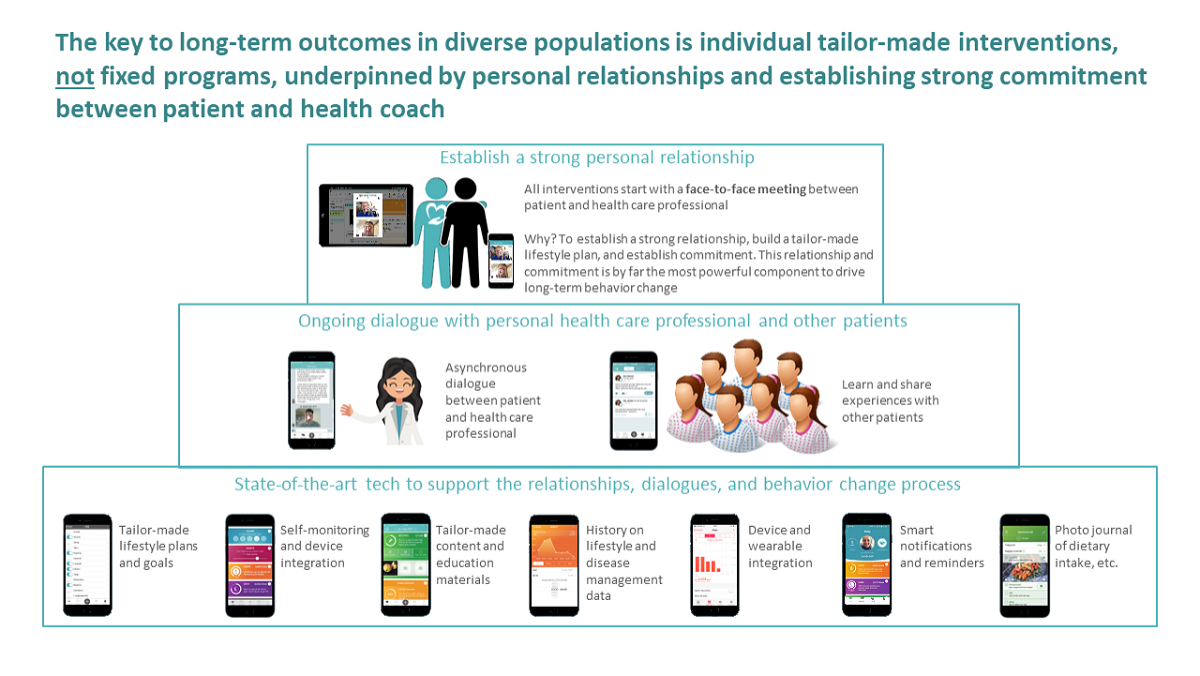
Patient experience using the eHealth intervention.

The patients registered their data on a smartphone or logged into a personal profile using the internet (see Figure 2).

**Figure 3:**
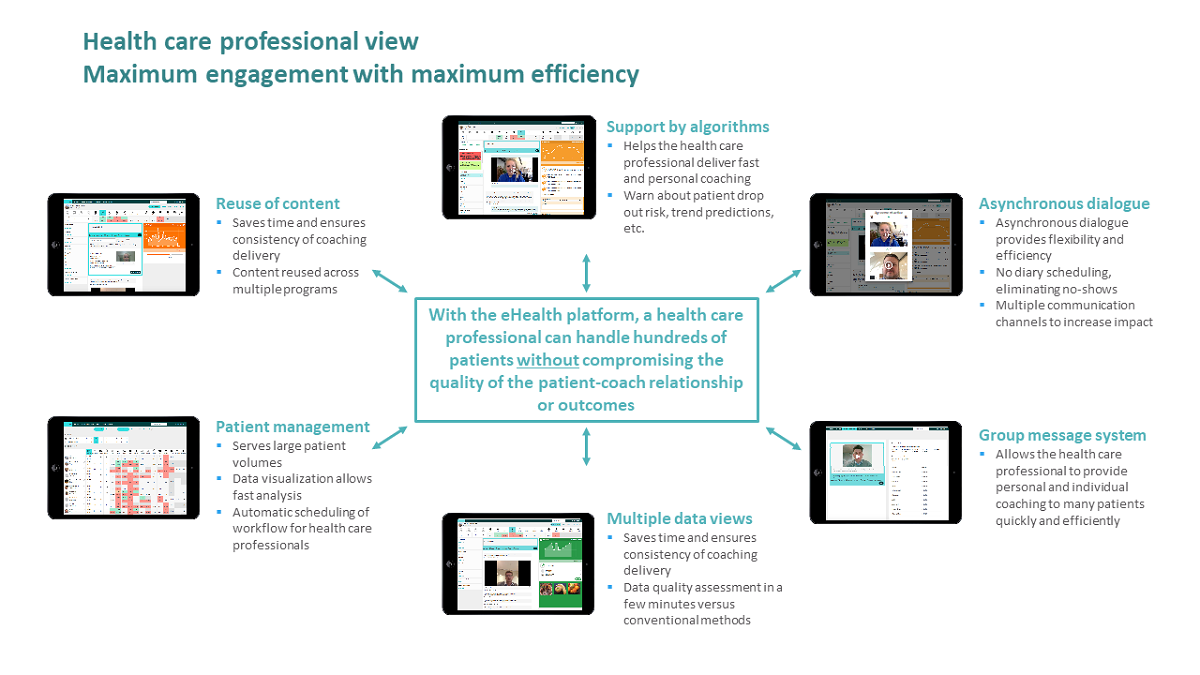
Healthcare professionals experience using the eHealth intervention.

The patients filled in a daily record as well as their comments, concerns, and questions to the municipal HCP, who had access to the participant profiles through a control panel. The municipal HCP provided individual asynchronous online consultation according to the patient’s needs based on the patient’s own registrations. The municipal HCP encouraged and praised goal attainment and sought to maintain the patient motivation. Within the first 3 months, patients were guided by the municipal HCP once every week. In the following 2 months, consultations were provided every second week. After this, guidance took place monthly until 12 months. The following year, the patient proceeded to the retention phase, receiving quarterly consultations described in Figure 3.

### Data Analysis

Outcome data were pooled across municipalities, and average findings were reported. We used a descriptive statistics approach to summarize characteristics of the study population. We reported means and SDs. We estimated weight change among diabetes patients in the periods of up to 6, 9, and 12 months. To examine the intervention impact on weight change, we used ordinary least square regression, including age, gender, and initial BMI as confounders. We investigated the effect of potential explanatory variables: number of messages sent by the patient, number of posts written in the forum, and engagement rate, which is the percentage of weeks where a patient actively uses the app out of the total weeks in the intervention. Applying *t* test and analysis of variance (ANOVA) tests, we compared weight change between male and female patients as well as across 3 age groups: <40 years, 40-59 years, and ≥60 years. Statistical significance was inferred at a 2-tailed *P*<.05. All analyses were completed using Stata version 14.1 (Stata, College Station, TX, USA).

**Table 1:** *Template for intervention description and replication* checklist for the electronic health lifestyle intervention.

TIDieR^a,b^ checklist item		Description	
What?		The health care professionals (HCPs) received training in setting SMART^c^ goals and digital coaching. Patients receive 1 or 2 personal meetings (face-to-face or digital) with the HCP, followed by asynchronous Web-based consultations based on dialog by means of short message service text message or video. The consultations addressed the patient’s registrations, goal setting, and questions regarding diet, exercise, and lifestyle plan and took chronic diseases into consideration. The LIVA app is set up with short explanations on different functions and notifications and reminders to the patients to register and give feedback on the health coaching. The sessions provide the user with information in relation to their status, specific focus on goals, and recommendations on how to improve their behaviors.	
eHealth coaching sessions		Included BCT^d^ from CALO-RE^e^ taxonomy (hereafter referred to as BCT): provide information on consequences of the behavior in *general* and *to the individual*, goal setting: behavior and outcome, action planning, barrier identification or problem solving, set graded tasks, prompt review of behavioral goals, prompt review of outcome goals, prompt rewards contingent on effort or progress toward behavior, prompting generalization of a target behavior, and provide feedback on performance.	
**Goals and inputs**		The goals and inputs described underneath are available to the patient, who can choose his or her focus area, set specific concrete goals, and keep record of specified behaviors by reporting on them on a daily, weekly, or monthly basis. This allows the user and the HCP to follow progress or setbacks as the numbers and registrations get visualized with graphs and curves. All advices from the HCP follow national guidelines from the Danish National Board of Health.	
	Dietary goals and plans		Dietary goals and plans can be set at many different levels from simple changes aiming at changing 1 meal a day to more complex changes aiming at a completely new diet composition to remedy digestion problems.
	Physical activity goals and plans		Goal setting and recording of type and time for executing any given physical activity. The user receives advice and/or video on activities in a variety of contexts to foster physical activity as a more integrated part of the person’s life (BCT: provide instruction on how to perform the behavior, prompting generalization of a target behavior, and relapse prevention or coping planning).
	Life goals		Goals on a healthy, joyful life as the patient sees it, for example, daily life with less stress, stronger social bonds with friends and family, and coping skills for diseases.
	Weight-input		Set current weight and goal for a lower or higher weight and register new measurements on a daily, weekly, or monthly basis.
	Steps-input		When downloading the app, the user can accept that their information on steps recorded on a smartphone are imported directly, and tailored messages on progress toward a set goal appear simultaneously (BCT: teach to use prompts or cues).
	Pain, sleep, and mood-input		Give daily feedback on pain, sleep, and mood, which can affect the ability to perform a given behavior (BCT: relapse prevention or coping planning).
	Smoking-input		Set goals to bring down the number of cigarettes smoked on a daily basis, leading to cessation.
	Blood glucose, cholesterol, and lung capacity-input		Keeping a record of specified measures expected to be influenced by the different behavior changes addressed. In LIVA, this includes blood glucose, cholesterol, and lung capacity. (BCT: prompt self-monitoring of behavioral outcome and provide information on consequences of the behavior in *general* and *to the individual*).
Forum		Online forum where the users can exchange knowledge, gain social support, and build new relationships; the health coach can add advices to the forum users (BCT: plan social support or social change).	
Who provided?		Health professionals with basic training as nurses, physiotherapist, dieticians, and occupational therapists were performing the health coaching.	
How?		Individually delivered via the app or Web.	
Where?		Initial personal meeting in the health centers or digital. Then solely Web-based delivery.	
When and how much?		The initial consultations with a health coach is estimated to last approximately 45 to 60 min. The following asynchronous eHealth coaching sessions were carried out once weekly in the first 3 months and then for maintenance every third week for the last 9 months. Hereafter, the patient can receive 2 eHealth coaching sessions and use LIVA as a personal behavioral change tool. (BCT: use of follow up prompts).	
Tailoring		Every patient received personal eHealth coaching sessions from their designated health coach. The feedback given was based on the patient’s inputs on LIVA.	

^a^TIDieR: *template for intervention description and replication.*

^b^On the basis of the study by Hoffmann et al [19].

^c^SMART: specific, measurable, agreed upon, realistic, and time-based goals.

^d^BCT: behavior change technique.

^e^CALO-RE: Coventry, Aberdeen, and London-Refined taxonomy [20].

## Results

### Patients at Baseline

On average, diabetes patients who participated in the program for 90 days or longer were obese at baseline, with 57 out of 103 (55.3%) being females. The study population has on average been in the intervention for 220 days (7.3 months; Table 2).

### Observed Weight Change

**Figure 4:**
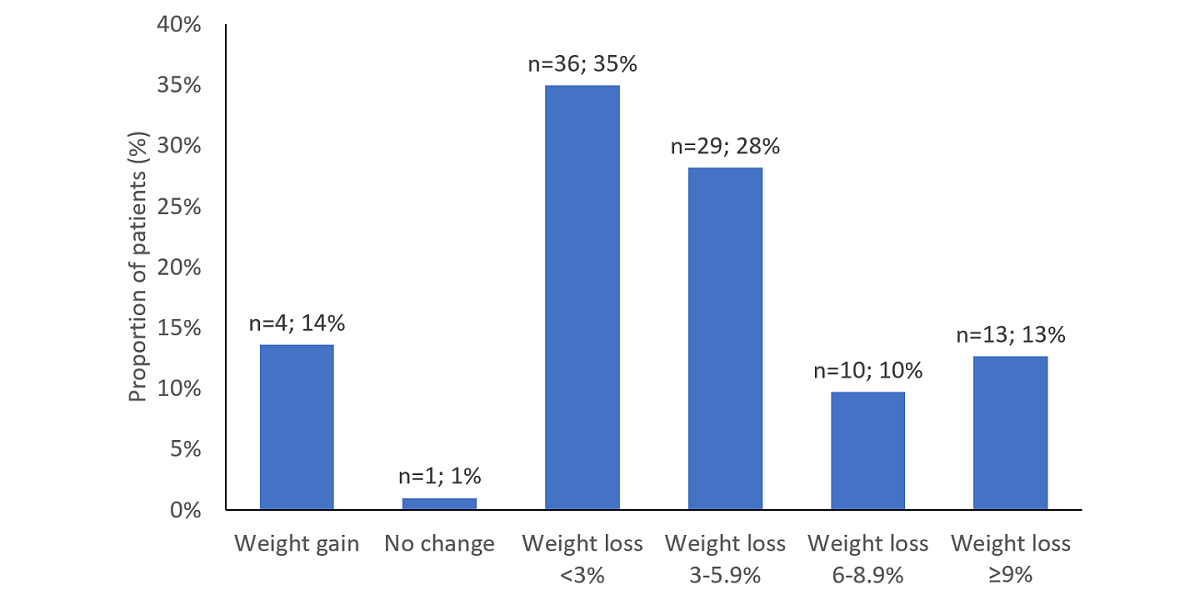
Observed weight change among the diabetes patients.

Majority of the study population, 88 out of 103 participants (85.4%), lost weight, whereas 15 patients (15/103, 14.6%) maintained or gained weight (Figure 4).

According to the examined data, on average, individuals with diabetes reduced their weight by 4.78 kg or 4.3% of their initial body mass, which corresponds to a 1.58-point change in BMI. Female patients lost 4.22% of the initial body mass, whereas male patients reduced their weight by 4.41% (Table 3).

**Table 2:** Baseline characteristics of the study population.

Characteristics	Statistics
Individuals (n)	103
Age (years), mean (SD)	55.6 (10.8)
Female, n (%)	57 (55.3)
Weight (kg), mean (SD)	106.8 (18.8)
Body mass index (kg/m^2^), mean (SD)	36.0 (5.2)
Duration (days), mean (min, max)	219.9 (92, 365)

**Table 3:** Weight change during the intervention according to the observation period (duration).

Characteristics		Duration (90-365 days)	Duration (90-179 days)	Duration (180-269 days)	Duration (270-365 days)
Individuals (n)		103	97	54	39
Female, n (%)		57 (55.3)	53 (55)	31 (57)	21 (54)
Duration (days), mean (min, max)		219.9 (92, 365)	148.8 (92, 179)	240.5 (182, 269)	330.5 (273, 365)
Weight change (kg), mean (SD)		−4.78 (6.67)	−4.31 (5.9)	−6.14 (7.92)	−6.78 (8.1)
**Weight change (% of initial weight), mean (SD)**		−4.3 (5.93)	−3.9 (5.34)	−5.56 (6.93)	−6.27 (7.64)
	Weight change in female	−4.22 (6.83)	−0.91 (6.09)	−5.96 (8.03)	−6.78 (9.12)
	Weight change in male	−4.41 (4.63)	−3.9 (4.33)	−5.02 (5.23)	−5.68 (5.67)
Body mass index change (kg/m^2^), mean (SD)		−1.58 (2.24)	−1.43 (2.0)	−2.05 (2.67)	−2.24 (2.67)

**Figure 5:**
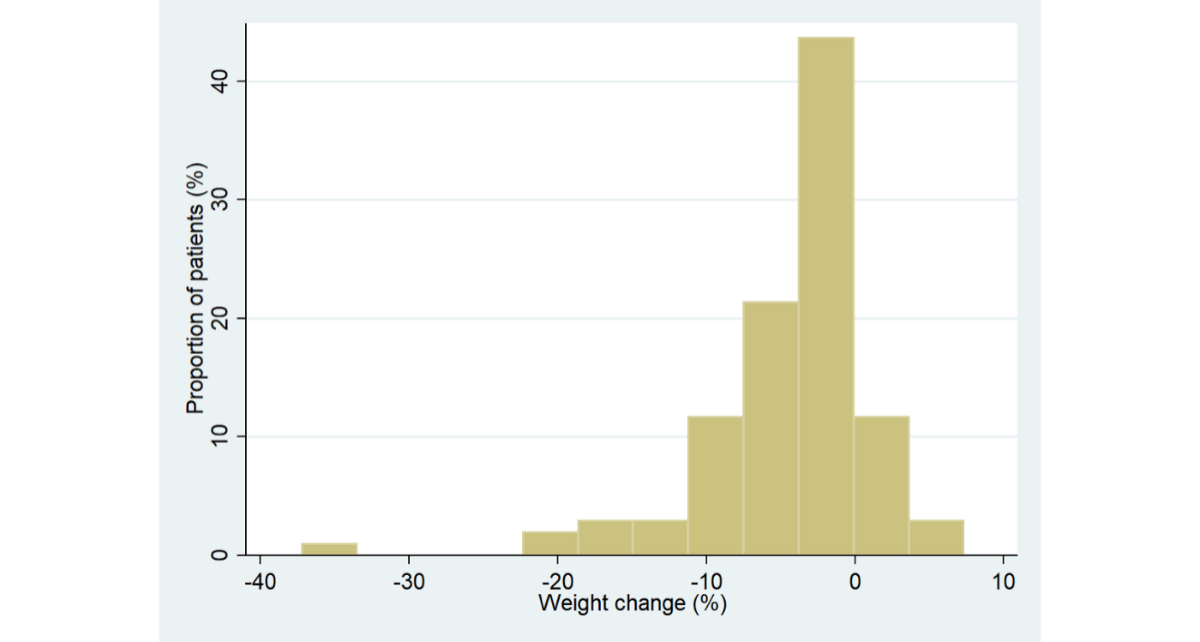
Distribution of percentage weight change among the diabetes patients.

Figure 5 illustrates the distribution of percentage weight change among the diabetes patients, where negative numbers indicate weight loss and positive numbers indicate weight gain.

**Figure 6:**
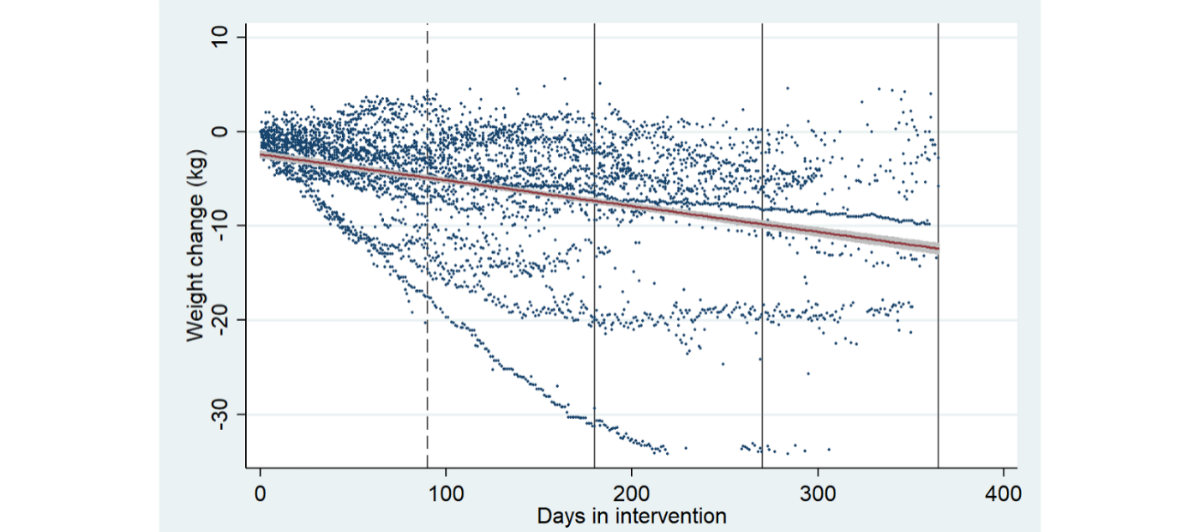
Weight change among the diabetes patients in intervention. Each dot represents a weight change estimated from the weight parameters registered by each diabetes patient. The red line with grey area illustrates prediction from a linear regression of weight change on days in intervention, including the CIs.

The weight change of those individuals who have registered their parameters up to 180 days was −3.9%, for those up to 270 days was −5.56%, and for those up to 365 days was −6.27% (Table 3). Figure 6 illustrates the weight change in kilos per person per day.

### Difference Between Patient Groups

The mean weight change among male patients was –5.04 kg and among female patients was –4.56 kg; the difference of 0.48 kg between the gender groups was not significant (*P*=.72). The observed mean weight change among patients older than 40 years (N=9) was −4.7 kg, 40 years to 59 years (N=56) was −4.9kg, and 60 years or older (N=38) was −4.6 kg. We applied 1-way ANOVA to examine mean weight change between the 3 age groups and found that the difference observed between the age groups was insignificant (*P*=.99).

### Impact of Intervention on Weight Change

The results of regression analysis indicate that participation in intervention has a significant impact on weight change, where time in intervention is associated with weight loss, implying that an extra day in intervention corresponds to 16 g weight loss (*P*=.02) or about 480 g per month (Table 4).

The additional explanatory variables were investigated with regard to the impact on weight change (kg). Table 5 presents the outcomes of the linear regression, where number of forum posts and engagement rate (%) are associated with weight loss, but results are statistically insignificant (*P*>.10).

**Table 4:** Results from regression analyses for prediction of weight change (kg). Regression model summary: N=103; *R*^2^=.108; adjusted *R*^2^=.071.

Explanatory variable	Regression coefficient	*P* value
Time	−0.016	.02
Baseline body mass index	−0.280	.03
Age	−0.008	.90
Gender	0.519	.69
Constant	8.503	.29

**Table 5:** Results from regression analyses for prediction of weight change (kg). Regression model summary: N=103; *R*^2^=.120; adjusted *R*^2^=.055.

Explanatory variable	Regression coefficient	*P* value
Time	−0.013	.03
Baseline body mass index	−0.252	.06
Age	−0.018	.78
Gender	1.079	.43
Sent messages	0.017	.69
Forum posts	−0.131	.29
Engagement	−0.024	.50
Constant	8.503	.29

## Discussion

### Principal Findings

Results show an average weight loss of 4.8 kg corresponding to 4.3% of initial body weight over a mean period of approximately 7 months. According to regression analysis, the weight loss is significant over time (*P*<.05). We found that time spent in intervention was the main driver for weight loss. Gender and age did not significantly influence the outcomes, indicating that the intervention effect is not dependent on traditional demographic characteristics, as often anticipated in lifestyle interventions [21].

Despite the anticipated impact of engagement in the app along with forum activity and messaging frequency on weight loss, the insignificant results indicate that motivation within the intervention is not equal to activity and engagement in the digital device. Further research based on a larger sample size would improve the results.

Haste et al found an average weight loss of 5.4 kg for the ones who completed a 12-month Web-based weight loss intervention for men with type 2 diabetes in a previous Web-based version of the collaborative eHealth intervention in a pilot randomized controlled trial in a municipality setting [6]. Other eHealth coaching programs among diabetes patients have found weight reductions of 6.8% to 7.5% of body weight among completers after 6 to 12 months [22-24]. A review concluded a mean reduction in body weight of 3.73 kg after 12 months among 13 studies, analyzing the effect of BCTs on diet and physical activity in type 2 diabetes [25]. This indicates that the validity of the trends observed is promising, especially given the modest investment compared with traditional lifestyle change or exercise interventions.

This study evaluates the effects associated with a real-life eHealth lifestyle coaching intervention (LIVA) in 8 Danish municipalities. The observational design allows for data to reflect a real-life setting based on a substantial number of observations; results show clear tendencies of significant self-reported weight reduction among diabetes patients.

Within the scope of this study, we conducted an extensive review of available literature that observes the impacts of weight reduction among the obese diabetes patients on the costs of diabetes [26-31]. Following the literature review, we expect that a 1% reduction in weight among diabetes patients corresponds to 3.1% decrease in societal costs of diabetes in Denmark, which were previously estimated by Sortsø et al [32]. The average weight loss of 4.3% among the diabetes patients within the cohort has a potential to reduce the annual diabetes costs of a single diabetes patient by 13.33%, corresponding to Euro 2676 savings per diabetes patient per year, compared with the no-intervention scenario (the costs of intervention were not included). Subtracting the implementation and running costs of the intervention, the implementation was found to be cost-effective in a municipal perspective already after 1 year of implementation [33].

Preventive strategies within diabetes have gained ground the past decade, for example, the Diabetes Prevention Program in the United States [22,24,34]. The National Health Service also launched a National Diabetes Prevention Program (NDPP) in 2016, which covered 75% of the nation in April 2017 [35,36]. These programs were initiated based on the expectation that lifestyle change among people at risk of or early in their diabetes will be effective in reducing the incidence of diabetes as well as late complications, thereby reducing costs. The economic assessment of the NDPP is undertaken from a 20-year perspective because of the disease structure of diabetes with risk of complications, and hence costs, increasing over time [37].

### Limitations of the Study

Self-reported data are always subject for reporting bias. Other studies have, however, shown that self-reported data in Web-based eHealth solutions are valid [38]. As we observe a weight difference as our outcome, we assume reporting bias to be equal for both baseline and end data. An objective measurement and improved registration of comorbidities could strengthen the data and allow the distribution of diabetes patients across complication groups.

This study reflects a natural experiment in the sense that we observe a running program implemented in a real-life municipal setting. This is a strength as well as a limitation in relation to disentangling the effect of the program. In a longer follow-up, analysis with more observations within municipalities would provide valuable insights.

Long-term evidence on sustainability of results is needed within this area. Hence, a register-based study with observation of actual costs of participants over a longer follow-up period and subsequent comparison with control population would provide important new knowledge. Furthermore, as we observe different effects of the program across municipalities, there is a need for in-depth analysis within each municipality to investigate what is decisive for the effect.

### Conclusions

We found that the collaborative eHealth tool significantly reduced weight among diabetes patients, on average 4.3% of the initial body mass with potential substantial cost savings. This study is based on a study population consisting of 103 individuals with diabetes, and it brings forward evidence of a positive effect of a running municipal secondary preventive offer that targets diabetes patients. Our study establishes a framework for further evaluation of eHealth tools where new data from longer follow-up can be examined to strengthen conclusions from this study.

### Perspectives and Implications

The findings in this study are of relevance for all HCPs working with primary and secondary prevention of diabetes. Collaborative eHealth lifestyle coaching tools offer promising new opportunities for successfully changing diabetes patients’ lifestyles. However, much remains unknown in this new era of eHealth possibilities. The results presented in this study are of importance, stressing that with a relatively modest personal resource investment, a collaborative eHealth lifestyle coaching tool can enable tailored coaching, resulting in significant weight losses among diabetes patients. We believe that these preliminary results show promising tendencies; however, there is a way yet to demonstrate sustainability of the weight loss attained as well as specific cost savings. We will investigate these issues along with long-term data arriving.

## Abbreviations

ANOVA: analysis of variance
ApHER: Institute of Applied Economics and Health Research Aps
BCT: behavior change technique
BMI: body mass index
eHealth: electronic health
HCP: health care professional
